# Feline Leishmaniosis in Northwestern Italy: Current Status and Zoonotic Implications

**DOI:** 10.3390/vetsci8100215

**Published:** 2021-10-02

**Authors:** Ehab Kotb Elmahallawy, Stefania Zanet, Marco Poggi, Khalaf F. Alsharif, Ahmad Agil, Anna Trisciuoglio, Ezio Ferroglio

**Affiliations:** 1 Department of Zoonotic Diseases, Faculty of Veterinary Medicine, Sohag University, Sohag 82524, Egypt; 2 Department of Pharmacology, Faculty of Medicine, University of Granada, 18002 Granada, Spain; aagil@ugr.es; 3 Department of Veterinary Sciences, University of Turin, Via Leonardo da Vinci, 44, Grugliasco, 10095 Torino, Italy; stefania.zanet@unito.it (S.Z.); marco.poggi@centroveterinarioimperiese.com (M.P.); anna.trisciuoglio@unito.it (A.T.); 4 Department of Clinical Laboratory Sciences, College of Applied Medical Sciences, Taif University, P.O. Box 11099, Taif 21944, Saudi Arabia; alsharif@tu.edu.sa

**Keywords:** *Leishmania infantum*, cats, western blot, PCR, Italy

## Abstract

Leishmaniasis remains one of the major neglected tropical diseases. The epidemiological profile of the disease comprises a wide range of hosts, including dogs and cats. Despite several studies about feline Leishmaniosis, the role of cats in disease epidemiology and its clinical impact is still debated. The present study raises awareness about the impact of leishmaniasis in cats from an endemic region in of Northwestern Italy (Liguria). A total number of 250 serum and 282 blood samples were collected from cats, then assessed for *Leishmania infantum* (*L. infantum*) serologically using western blot (WB) and molecularly using polymerase chain reaction (PCR). We also tested the association of *Leishmania* infection with some infectious agents like haemotropic *Mycoplasma*, *Feline immunodeficiency virus* (FIV) and *Feline leukemia virus* (FeLV) together with the hematobiochemical status of the examined animals. Interestingly, all tested animals were asymptomatic and out of 250 examined serum samples, 33 (13.20%) samples (confidence interval (CI) 95% 9.56–17.96%) were positive at WB for *L. infantum*, whereas of the 282 blood samples, 80 (28.36%) returned a positive PCR (CI 95% 23.43–33.89%). Furthermore, there was a statistical association between PCR positivity for *L. infantum* and some hematological parameters besides FIV infection as well as a direct significant correlation between *Mycoplasma* infection and WB positivity. Taken together, the present findings report high prevalence of *L. infantum* among cats, which reinforces the significance of such positive asymptomatic animals and confirms the very low humoral response in this species. In addition, the laboratory values provide evidence that infection by the parasite is linked to alteration of some hematological parameters and is correlated to some infectious agents. These data are of interest and suggest future research for accurate diagnosis of such zoonosis.

## 1. Introduction

Leishmaniasis is a group of neglected tropical diseases caused by an opportunistic intracellular protozoan of the genus *Leishmania* and transmitted to humans by the bite of female sandflies [1,2,3]. Among 15 well-recognized species of subgenus *Leishmania*, 13 species are zoonotic [3,4]. Nowadays, about 12 million people infected in 98 countries, and 350 million people are at risk [3,5,6]; the distribution of disease has traditionally been linked to tropical and subtropical regions besides being endemic in many areas worldwide such as the Mediterranean basin, East Africa and South America [1,3]. Three main forms of the disease are known: cutaneous leishmaniasis (CL), mucocutaneous leishmaniasis (MCL), and disseminated visceral infection (VL), with fatal prognosis in absence of treatment [7,8]. The latter form, VL, is mainly caused by the *Leishmania donovani* (*L. donovani*) complex (Africa, India, and Asia) and by *L. chagasi* (*L. infantum*) in South America and the Mediterranean area [8,9]. In the Mediterranean basin, *L. infantum* is named zoonotic visceral leishmaniasis (ZVL) where dogs are the main reservoir host [10,11].

The last decade has witnessed extraordinary progress in the spread of the infection to new areas, in particular due to global change and/or movements of hosts and vectors [12,13,14]. This has resulted in the appearance of new endemic foci for the disease, combined with increasing reports of new reservoirs and transmission dynamics. Clearly, exploring new reservoirs and understanding the transmission dynamics has spurred great interest amongst the scientific community, with the goal of understanding the roles of several animal species as potential reservoirs for infection [1]. This has led to the necessity of more surveying and searching for novel drug targets aiming to construct effective control measures adopted to eliminating transmission and preventing new epidemics [7,15,16].

In accordance with the epidemiological profile of ZVL, it includes humans, rodents, domestic and wild animals; however, dogs are considered the most important reservoirs in the domestic environment [17]. The role of cats as reservoirs of *Leishmania* is still controversial and has never been clarified due to lack of xenodiagnosis trials; however, cats remain secondary reservoirs, rather than accidental ones [4,18,19,20,21]. Despite the alarming increase in the number of reports about leishmaniasis in cats, there is still a lack of information about the actual role played by cats in maintaining the epidemiological pattern of the disease, which may be due to a high degree of natural resistance, except in some immunosuppressed conditions like feline virus infection and cancer [20,22,23]. Given the fact that *Leishmania*-infected cats are most frequently asymptomatic or manifest symptoms as light cutaneous lesions such as skin ulcers, feline clinical leishmaniasis is relatively rare, though cats can contract the infection [24]. Among other *Leishmania* species, *L. infantum* and *L. donovani* are the most common species in domesticated cats in the Mediterranean area and Middle East [25], while *L. major*/*L. donovani (sensu lato)* hybrid parasites were detected in cats in mainland Portugal and Spain [26,27]. On the Other hand, *L. chagasi*, *L. braziliensis*, *L. amazonensis* and *L. mexicana* are the major species in Central and South America [28,29,30,31]. Association of some opportunistic pathogens like *Feline immunodeficiency virus* (FIV) and FeLV with infection may enhance or activate the infections, which may result in the appearance of some clinical signs [24,32]. Taking into account the frequency of asymptomatic infection in cats, diagnosis of *Leishmania* infection is somewhat complex. Among the available diagnostic techniques, polymerase chain reaction (PCR) is considered a highly efficient, noninvasive tool for diagnosis and follow-up of the disease [33]. Likewise, clinical VL is usually accompanied by high antibody titers, which has led to the widespread use of several serological techniques with the aim of replacement or complementation of parasitological diagnosis [34]. The present study explored the occurence of *L. infantum* in cats from a highly endemic area in northwestern Italy. We also aimed to test the association between *Leishmania* and some infectious agents like haemotropic *Mycoplasma* species and Retroviruses including FIV and FeLV. Hematobiochemical alterations were also considered as variables associated with infection.

## 2. Material and Methods

### 2.1. Ethical Considerations

Our study complied with current Italian legislation on research and received ethical approval from the Department of Veterinary Sciences (Local ethical approval), University of Turin, which complies with all relevant CORDIS (European Commission).

### 2.2. Study Population

A total number of 346 domestic cats (*n* = 250 serum and *n* = 282 blood samples) from Liguria, a coastal region located in Northwestern Italy, were routinely sampled during veterinary clinic visits and underwent screening. The full details of the study cohort are shown in the Supplementary Materials (Table S1). To investigate *Leishmania* infection, 2 mL of blood was collected from the animal (through jugular vein puncture) in a clean sterile tube, both with Ethylene diamine tetraacetic acid (EDTA) and without anticoagulant. Samples were then transported to the laboratory of the division of parasitology, Department of Veterinary Sciences, University of Turin, Italy where the serum samples were centrifuged and stored at −80 °C until testing and the blood samples were processed to perform further tests and the experimental steps.

### 2.3. Clinical and Laboratory Diagnosis

Cats were examined clinically for any lesions together with routine hematochemical examination. For the complete blood count (CBC), a Pentra60 Horiba ABXTM (Horiba ABX; Irvine, CA, USA) was used to count the blood cells, whereas for biochemical a MIRA Plus Horiba ABXTM (chemical liquid, Horiba ABX; Irvine, CA, USA) and for serum protein electrophoresis, an SAE-NT of Chemetron was used. The haematochemical parameters included: white blood cell count (WBC), monocytes (MON), neutrophils (NEU), eosinophils (EOS), lymphocytes (LYM), red-blood cell count (RBC), hemoglobin (Hb), hematocrit (HCT), platelets count (PLT); albumin (ALB), total protein (TP), creatinine (CREA), blood urea nitrogen (BUN), alanine transaminase (ALT), aspartate aminotransferase (AST), alkaline phosphatase (ALP), cholesterol (CHOL), triglycerides (TG), glucose (GLU), calcium (Ca), potassium (K) and phosphorus (PHOS). Serum samples were also serologically screened by western blotting, while molecular detection was carried out using PCR with the blood samples.

### 2.4. Western Blotting and SDS-Polyacrylamide Gel Electrophoresis

Serum samples were screened by WB using *L. infantum* promastigotes as antigen, according to the method previously described for different species, including cats [35,36]. Briefly, Two hundred micrograms (1 mg/mL) of promastigotes lysate was run on a 12% polyacrylamide gel (SDS–PAGE). Molecular weight markers were used on a separate track. Fractioned proteins were electroblotted (350 mA, 1 h) onto nitrocellulose sheets that were saturated (3 h) with 3% bovine serum albumin in Tris-Buffered Saline (TBS). Sera were diluted 1: 10 in TBS and incubated overnight at 4C in a Multiscreen Apparatus with reference positive and negative sera (Bio-Rad, Hercules, CA, USA). After washing three times with 0.05% Tween20-TBS, the secondary antibody (1:8000; horseradish peroxidase labelled goat anti-cat IgG (H + L); Novex by Life Tecnologies, Waltham, MA, USA) was incubated for 1 h. Then, the nitrocellulose membrane was washed three times, and anti-*L. infantum* antibodies were revealed using the ECL system (GE Healthcare, Chalfont St Giles, UK).

Serum samples were screened by WB using *L. infantum* promastigotes as antigen, according to the method previously described for different species, including cats [35,36]. Briefly, Two hundred micrograms (1 mg/mL) of promastigotes lysate was run on a 12% polyacrylamide gel (SDS–PAGE). Molecular weight markers were used on a separate track. Fractioned proteins were electroblotted (350 mA, 1 h) onto nitrocellulose sheets that were saturated (3 h) with 3% bovine serum albumin in Tris-Buffered Saline (TBS). Sera were diluted 1: 10 in TBS and incubated overnight at 4C in a Multiscreen Apparatus with reference positive and negative sera (Bio-Rad, Hercules, CA, USA). After washing three times with 0.05% Tween20-TBS, the secondary antibody (1: 8000; horseradish peroxidase labelled goat anti-cat IgG (H + L); Novex by Life Tecnologies, Waltham, MA, USA) was incubated for 1 h. Then, the nitrocellulose membrane was washed three times, and anti-*L. infantum* antibodies were revealed using the ECL system (GE Healthcare, Chalfont St Giles, UK).

The resulting bands were compared using as a marker Prestained Protein Molecular Weight Marker (Fermentas International Inc, Ontario, ON, Canada) and Biotinylated SDS PAGE Standards broad range (BioRad Laboratories, California, CA, USA). Samples were considered positive by WB when at least two bands of 169, 115, 66, or 33 kDa could be detected [36].

### 2.5. Preparation of Blood Samples and Extraction of DNA 

Total genomic DNA was extracted from 200 μL of whole blood using GenomeElute commercial kit under conditions suggested by the manufacturer (Sigma–Aldrich). The DNA was then stored in sterile DNAse- and RNAse-free microtubes and kept at −20 °C.

### 2.6. Qualitative Polymerase Chain Reaction (PCR) for Detection of Leishmania and Mycoplasma DNA

We adapted a previously described PCR protocol [33] that uses primers mRV1 5′ CTTTTCTGGTCCCGCGGGTAGG-3′ and mRV2 (5′-CCACCTGGCCTATTTTACACCA-3′) to amplify a 145 bp fragment present on the highly reiterated kDNA minicircle of *L. infantum*. The PCR reaction mixture (25 μL) contained ≈ 100 ng of DNA template, 2.5 μL 10X PCR buffer, 5 μL of Q Buffer, 2.5 UI of HotStarTaq DNA Polymerase (Qiagen, Milan, Italy), 0.5 μL of dNTPs mix (10 mM of each dNTP, Sigma-Aldrich, St. Louis, MO, USA), and 12.5 pmol of each primer. An initial denaturation step of 15 min at 95 °C was followed by 35 repeats of 15 min at 95 °C, 1 min at 60 °C, and 1 min at 72 °C, and a final elongation step of 10 min at 72 °C. To detect the presence of haemotropic *mycoplasmas*, we developed a multiplex PCR protocol targeting the 16 s rRNA gene. Species specific forward primers (MhfF 5′-TCTTTGGTTTCGGCCAAAGAT-3′, MhmF 5′-GCTTGATAGGAAATGATTAAGC-3′, and MtcF 5′-TCCTCCATCAGACAGAAGGGGGA-3′ for *Mycoplasma haemofelis, Candidatus Mycoplasma haemominutum, and Candidatus Mycoplasma turicensis*, respectively) were used together with a common reverse primer MycR 5′-GGGTATCTAATCCCATTGC-3′. The multiplex PCR was optimized in a final volume of 25 µL using Promega PCR Master Mix (Promega Corporation, WI, USA), together with 1 µM of primers MhfF and MtcF, 0.5 µM of MhmF, 2 µM of the reverse primer MycR and ≈100 ng of DNA template. The amplification included a 5 min denaturation step at 95 °C followed by 40 repeats of 1 min at 95 °C, 30 s at 60 °C, and 3 min at 72 °C and a final extension at 72 °C for 10 min. PCR fragment size was estimated by comparison with two molecular weight standards (control): PCR 100 pb Low Ladder and pBR 322 HaeIII Digest (Sigma–Aldrich) after electrophoresis on a 2% agarose gel. Gels were stained with MegaFluor kit (Euroclone, Milano, Italy) performed under the conditions suggested by the manufacturer and photographed on a Gel-Doc System (Bio-Rad). Quantity and quality of DNA were assessed using photometric UV-based measurement of nucleic acids (Thermo Scientific™ NanoDrop 2000, ThermoFisher, Waltham, MA, USA) and agarose gel electrophoresis. Internal positive (*L. infantum* DNA from cultured promastigotes) and negative (double distilled water) controls were included in each reaction.. Fifteen randomly selected positive PCR products were sequenced (Macrogen Inc., Amsterdam, The Netherlands) and the resulting sequences were compared to those available in GenBank. 

### 2.7. Detection of FeLV Antigen and FIV Antibody 

To test the correlation between Retroviral and *Leishmania* infection, 87 samples were tested for the presence of FeLV antigen and 89 samples for FIV antibody. Detection of FeLV antigen (p27) and FIV antibody was performed using immunochromatographic BVTTM until 2006. From 2007 onwards, ELISA Test Snap IDEXXTM commercial assay kit (SNAP^®^ FIV Antibody/FeLV Antigen Combo Test; IDEXX Laboratories, Westbrook, ME, USA) was used.

### 2.8. Data Analysis

To identify possible associations between *L. infantum* prevalence and seroprevalence, we used generalized linear mixed models in which the result of the PCR and WB was the dichotomous response variable. The potential explanatory variables (covariates) considered were: individual factors (breed, age, sex), and hemato-biochemical parameters (WBC, MON, NEU, EOS, LYM, RBC, Hb, HCT, PLT, ALB, TP, CREA, BUN, ALT, AST, ALP, CHOL, TG, GLU, Ca, K and PHOS). Concurrent infection with FeLV, FIV and Mycoplasma were also investigated as potential risk factors. The Variance inflation Factor (VIF) was used to test and avoid multicollinearity among predictors [37]. Variables identified as significant factors (*p* ≤ 0.05) in the first univariate analysis were selected for further testing by multivariate linear regression. Best model selection was performed using AIC (Akaike information Criterion), while the goodness-of-fit of the final model was assessed by computing the area under the curve (AUC) of the receiver operating characteristic plots. All statistical analyses were performed using R Statistical Software (Foundation for Statistical Computing, Vienna, Austria) [38].

## 3. Results

As previously shown, all cats underwent clinical examination for any lesion and they were asymptomatic. Of the 250 sera tested with WB, 33 samples tested positive (CI 95% 9.56–17.96%) for *L. infantum* with prevalence of 13.20%, while of 282 blood samples tested with PCR, 80 samples were positive for *L. infantum* with a prevalence of 28.37% (CI 95% 23.43–33.89%) (Table 1). It was possible to test a total of 186 cats by both PCR and WB. Of these, 10 tested positive by both PCR and WB (Table 1). Sequencing confirmed the specificity of the protocol used, as all the sequenced amplicons were identified as *L. infantum* (identity ≥ 98% to GenBank accession number: AB678348). 

On the other hand, 17 out of the 167 samples tested with PCR for various species of *Mycoplasma* (CI 95% 6.45–15.70%) were positive with a prevalence of 10.18%, including eight which were also positive for *Leishmania* using PCR and another three which were positive using WB and PCR. In addition, out of the 89 tested samples for FIV, 31 (34.83%) samples (CI 95% 25.75–45.17%) were positive, whereas of 87 test FeLV performed, 22 (IC 95% 17.33–35.33%) samples tested positive, for a prevalence of 25.29%. PCR positivity for *L. infantum* resulted in a positive association with higher values of BUN, NEU and with concomitant infection with FIV (*p* < 0.05), while there was a negative correlation with low values of HCT, RBC and Hb (*p* ≤ 0.05). The best fitting generalized linear model included FIV, BUN and RBC (AUC = 0.82, AIC = 181.75). For WB, our data evidenced only a direct significant correlation between *Mycoplasma* infection and the presence of anti-*L. infantum* antibodies. The characteristics of those variables which were significantly associated with PCR or WB results are summarized in Table 2.

## 4. Discussion

The present study reports a high prevalence of infection with *L. infantum*, either serologically or molecularly, in examined cats from an endemic area. Likewise, our data provide more evidence about the correlation between infection of cats with *L.* *infantum* as widely known opportunistic pathogen and concomitant infection with some other pathogens like FIV, FeLV and three feline hemoplasma species that include *Mycoplasma haemofelis*, *Mycoplasma haemominutum*, and *Mycoplasma turicensis*. To the best our knowledge, this study is the first epidemiological investigation performed on leishmaniasis in cats in this endemic area in Northwestern Italy (Liguria). Given the fact that leishmaniasis is not as common in cats as in dogs, some previous reports have explored the occurrence of feline leishmaniasis worldwide, especially in some countries where the zoonotic form of the disease is present [28,29,30,31,39,40,41,42]. Our present data report that all examined cats were asymptomatic, which is consistent with the hypothesis stating that leishmaniasis is mostly subclinical in cats [20,24]. However, some scant clinical cases were reported elsewhere with typical cutaneous signs, including ulcer crusted dermatitis and nodular lesions on the nose, lips, ears, and eyelids [43]. Other groups may develop chronic ulceration, located particularly on the head and limbs [43]. In rare reported cases of VL, the infected cases showed visceral involvement of the liver, spleen, lymph nodes and kidneys together with some cutaneous manifestations [4,20,44,45]. 

In Europe, several clinical cases of feline leishmaniasis have been described since 1911 to date in France, Greece, Switzerland, Spain, Italy, and Portugal, with very controversial results among serological and molecular methods [19,20,28,32,41,46,47,48,49]. In accordance with its prevalence in southern Europe, where the disease in dogs is endemic, several sero-epidemiological studies have shown a prevalence of *L.infantum* in cats ranging from 0.6% to 68%, with differences among countries or even within the same country, in particular Italy [18,40,41,46,47,48,50,51,52,53,54,55]. Diagnosis of *L. infantum* in combination with concurrent diagnosis of several opportunistic pathogens provides an important overview for better understanding the epidemiology of the disease. In this regard, western blotting is a core technique used to detect and quantify proteins that react with a specific antibody, among other available serological tests. Besides its high specificity and sensitivity, WB has shown greater sensitivity than immunofluorescence antibody test (IFAT) and Enzyme-linked immunosorbent assay (ELISA) in detection of infection with *Leishmania*, making it recommended mainly in doubtful cases [56,57,58]. In the present study, the prevalence of infection using WB yielded 13.20%. Nearly similar results have also been reported in some previous studies in the same country [52,59,60]. On the other hand, our present results are higher than previous studies in the same country; a previous study in Liguria and Tuscany, Italy recorded lower seroprevalence (0.9%) using IFAT [50]. Likewise, lower results were recorded in a nationwide survey of *L. infantum* in Italy using a combination of serological and molecular methods where the prevalence by serology was 3.3%, with a higher rate of cumulative prevalence (10.5%) in southern Italy than in the North (1.6%) [32]. In another study in southern Italy, a high cumulative serological prevalence was recorded using IFAT and molecular prevalence (25.8%) of *L. infantum* in cats [59]. Collectively, comparison of prevalence data even from the same contry is difficult, due to the differences among applied serological techniques which include either ELISA [18] IFAT [30,46,47,50,55,61], or direct agglutination test (DAT) [53,62]). This difference supports the use of WB in diagnosing both the clinical disease and subclinical infection [58,63,64,65]. Geographic location, habit changes and expansion range of sandfly vectors represent other factors that might influence the degree of endemicity of the disease [12,13,66,67,68], also taking into account that several serological studies have revealed that *Leishmania*-infected cats often develop a low level of humoral response or remain seronegative [47,50]. Furthermore, it should be borne in mind that different biological samples and PCR targets may affect the prevalence rate, which could be another explanation for the large variability in prevalence data reported so far [69,70].

Interestingly, polymerase chain reaction (PCR) is a widely accepted molecular tool for identification and quantification of *Leishmania* spp. in various tissues and body fluids in reservoir species or hosts [30,33,71]. Hence, several studies have recommended PCR as a highly efficient noninvasive tool for diagnosis and follow-up of the disease [72,73,74]. Regarding its prevalence in southern Europe, the positivity to *Leishmania* spp. by PCR in a previous studies ranged from 3% to 61%, explaining the possible influence of geographical location on the epidemiological pattern of the disease [32,47,75]. In the present study, we have reported positivity of 28.37% using PCR, which is similar to several previous studies in which the same method was applied [46]. In Southern Italy, a previous study detected a prevalence of infection (by PCR) of 25.8% [59]. On the other hand, lower results were reported in a previous nationwide survey in Italy for *L. infantum* using qPCR, where the overall cumulative prevalence was 0.8% [32]. In another study in cats in Northern Sardinia, Italy, molecular detection showed a prevalence rate of 5.5% for *L. infantum* in the population of tested cats [60]. As for sereoprevalence, previous reports of molecular prevalence in cats are subject to great variability among geographical areas [1,4,11,32]. 

The role of some other pathogens, viral infection such as FeLV, FIV and *Mycoplasma*, and their association to *Leishmania* infection, is still unclear and sometimes the results are controversial [50,76] As mentioned above, the occurrence of leishmaniasis in cats worldwide is usually asymptomatic; however, some of the affected cases were co-infected with FIV and/or FeLV, which may induce an impaired cellular immune response [20,77]. Based on our results, there was a statistical correlation between the positivity of sample to *L. infantum* using PCR and FIV, anemic status of an animal, blood urea nitrogen level, and neutrophilia, whereas using WB, there was a significant association between positivity to *L. infantum* and *Mycoplasma* infection. These data are consistent with several previous reports at both the national and international level [32,45,50,55,76,78,79,80]. Our data suggest a role played by some viruses like FIV as a retroviral infection in development of some opportunistic pathogens like *Leishmania* [7,32,51]. However, it should be borne in mind that the sole presence of FIV is not a sufficient marker to demonstrate immunodeficient status; this requires additional immunological tests. On the other hand, there was no a clear relationship between infected cats with FeLV and contraction of infection by *Leishmania*, which is consistent with several previous results [18,47,48,52,54,81]. The possibility of identifing a set of multiparametric infectious/haematochemical values (i.e., FIV, BUN and RBC, which were included in the best fitting model of infection in cats) could be used as predictive tool that could allow clinicians to consider *L. infantum* in their differential diagnosis. Taken into account, the resistance of a cat to *Leishmania* infection probably depends on genetic factors, and is not solely related to cell mediated immunity [20,82,83]. As shown, the use of direct (PCR) and indirect (WB) methods yielded discrepant results, which has allowed us to show the major advantages provided by the combined use of both methods for detection of the prevalence of infection in cats as compared to serology alone. Nevertheless, the reported prevalence rate highlights the role played by cats in the transmission of disease, and this suggests cats could be either primary or secondary reservoir host for *L. infantum*, consistent with previous reports that revealed contraction of infection by sandflies in cats naturally infected with *Leishmania* [84,85]. The explanation for this data lies not in the difference in sensitivity of the two methods, but rather in the fact that in some species resistant to infection, like cats, the activated immune response is particularly of cell mediated rather than humoral immunity [78,82]. Interestingly, our present data might contribute to the hypothesis suggesting that the cat predominantly has a Th1 response, as evidenced by the high number infections (positivity to PCR) compared to the number of subjects with circulating antibodies (positive to WB), and the limited occurrence of non-specific clinical signs [78,86]. In addition, our present data give more information about the association between *Leishmania* infection and both retroviruses and hemoplasmosis, which seem to play a role in the pathogenesis of the disease and even in susceptibility to infection [80]. 

## 5. Conclusions

Our present data reporting high prevalence of *L. infantum* in asymptomatic cats represent an important parameter to further take into account in epidemiological studies. Interestingly, our data may contributes to the hypothesis stating that cats may be a *Leishmania* reservoir, and suggest further studies to explore their role in maintaining the epidemiological foci of VL in the Mediterranean area. Furthermore, our data suggest performing routine and preferably combined (serological and molecular) testing for leishmaniasis in cats, especially in endemic areas, regardless of the presence or absence of clinical signs. Furthermore, the identfied set of multiparametric infectious/haematochemical values, mainly FIV, BUN and RBC, could be used as predictive tool for diagnosis of *L. infantum* in cats. Importantly, more strict alternative prophylactic strategies may be essential to reduce the risk of infection and promote identification of new models of leishmanial transmission. 

## Figures and Tables

**Table 1:** Results of polymerase chain reaction (PCR) and WB.

WB			
PCR	Negative	Positive	Total
negative	118	17	135
positive	41	10	51
Total	159	27	186

**Table 2:** *L. infantum* PCR results were found to be significantly associated with Feline Immunodeficiency Virus (FIV) infection, with higher values of Blood Urea Nitrogen (BUN) and Neutrophils (NEUTR), and with low values of Hematocrit (HCT), Red blood cells (RBC), and Hemoglobin (Hb). Western blot (WB) positivity was instead positively associated with concurrent *Mycoplasma* spp. infection. The data of each covariate are summarized in the table.

WB Associate Covariates						
Qualitative variables		Number of WB positive cats	Number of WB negative cats	*p*	odds ratio (OR)	Confidence interval (CI 95%)
*Mycoplasma* spp.	Pos	4	5	0.02	4.39	1.078–18.63
	Neg	15	84			
PCR associated covariates						
Qualitative variables		Number of PCR positive cats	Number of PCR negative cats	*p*	OR	CI 95%
FIV	Pos	23	18	0.003	3. 83	1.56–9.41
	Neg	12	36			
Quantitative variables		Mean value (min-max)		*p*	OR	CI 95%
BUN	PCR pos	119 (18–600)		0.047	1.78	1.38–6.27
	PCR neg	93 (5.59–583)				
NEUTR	PCR pos	10.84 (0.21–37.5)		0.050	2.01	1.03–9.58
	PCR neg	8.30 (0.33–22.12)				
HCT	PCR pos	18.37 (4.6–43.4)		0.032	0.86	0.05–0.46
	PCR neg	24.94 (4.2–73.4)				
RBC	PCR pos	4.04 (0.95–10.12)		0.048	0.78	0.01–0.95
	PCR neg	5.34 (1.04–11.65)				
Hb	PCR pos	6.15 (1.6–14.3)		0.050	0.81	0.04–0.93
	PCR neg	8.18 (1.7–27.1)				

## Data Availability

The data that support the findings of this study is contained within the article.

## Supplementary Materials

The following are available online at https://www.mdpi.com/article/10.3390/vetsci8100215/s1, Table S1: The full details of the study cohort for each of the enrolled cat’s sex, age, breed and living habits.

## References

[1] Clem A. (2010). A current perspective on leishmaniasis. J. Glob. Infect. Dis..

[2] Romero H.D., Silva Lde A., Silva-Vergara M.L., Rodrigues V., Costa R.T., Guimaraes S.F., Alecrim W., Moraes-Souza H., Prata A. (2009). Comparative study of serologic tests for the diagnosis of asymptomatic visceral leishmaniasis in an endemic area. Am. J. Trop. Med. Hyg..

[3] WHO (2010). Control of the Leishmaniases.

[4] Gramiccia M., Gradoni L. (2005). The current status of zoonotic leishmaniases and approaches to disease control. Int. J. Parasitol..

[5] Alvar J., Velez I.D., Bern C., Herrero M., Desjeux P., Cano J., Jannin J., den Boer M., WHO Leishmaniasis Control Team (2012). Leishmaniasis worldwide and global estimates of its incidence. PLoS ONE.

[6] Bates P.A., Rogers M.E. (2004). New insights into the developmental biology and transmission mechanisms of *Leishmania*. Curr. Mol. Med..

[7] Elmahallawy E.K., Sampedro Martinez A., Rodriguez-Granger J., Hoyos-Mallecot Y., Agil A., Navarro Mari J.M., Gutierrez Fernandez J. (2014). Diagnosis of leishmaniasis. J. Infect. Dev. Ctries..

[8] Herwaldt B.L. (1999). Leishmaniasis. Lancet.

[9] Chappuis F., Sundar S., Hailu A., Ghalib H., Rijal S., Peeling R.W., Alvar J., Boelaert M. (2007). Visceral leishmaniasis: What are the needs for diagnosis, treatment and control?. Nat. Rev. Microbiol..

[10] Quinnell R.J., Courtenay O. (2009). Transmission, reservoir hosts and control of zoonotic visceral leishmaniasis. Parasitology.

[11] Dujardin J.C., Campino L., Canavate C., Dedet J.P., Gradoni L., Soteriadou K., Mazeris A., Ozbel Y., Boelaert M. (2008). Spread of vector-borne diseases and neglect of Leishmaniasis, Europe. Emerg. Infect. Dis..

[12] Maroli M., Rossi L., Baldelli R., Capelli G., Ferroglio E., Genchi C., Gramiccia M., Mortarino M., Pietrobelli M., Gradoni L. (2008). The northward spread of leishmaniasis in Italy: Evidence from retrospective and ongoing studies on the canine reservoir and phlebotomine vectors. Trop. Med. Int. Health.

[13] Ferroglio E., Romano A., Dettoni F., Trisciuoglio A. (2010). Distribution of Phlebotomus perniciosus in North-Italy: A study on 18S rDNA of phlebotomine sand flies. Vet. Parasitol..

[14] Caminade C., McIntyre K.M., Jones A.E. (2019). Impact of recent and future climate change on vector-borne diseases. Ann. N. Y. Acad. Sci..

[15] Elmahallawy E.K., Agil A. (2015). Treatment of leishmaniasis: A review and assessment of recent research. Curr. Pharm. Des..

[16] Elmahallawy E.K., Jimenez-Aranda A., Martinez A.S., Rodriguez-Granger J., Navarro-Alarcon M., Gutierrez-Fernandez J., Agil A. (2014). Activity of melatonin against *Leishmania infantum* promastigotes by mitochondrial dependent pathway. Chem. Biol. Interact..

[17] Claborn D. The Epidemiology and Ecology of Leishmaniasis.

[18] Solano-Gallego L., Rodriguez-Cortes A., Iniesta L., Quintana J., Pastor J., Espada Y., Portus M., Alberola J. (2007). Cross-sectional serosurvey of feline leishmaniasis in ecoregions around the Northwestern Mediterranean. Am. J. Trop. Med. Hyg..

[19] Miro G., Ruperez C., Checa R., Galvez R., Hernandez L., Garcia M., Canorea I., Marino V., Montoya A. (2014). Current status of *L. infantum* infection in stray cats in the Madrid region (Spain): Implications for the recent outbreak of human leishmaniosis?. Parasit. Vectors.

[20] Mancianti F. (2004). Feline leishmaniasis: What’s the epidemiological role of the cat?. Parassitologia.

[21] Maia C., Campino L. (2011). Can domestic cats be considered reservoir hosts of zoonotic leishmaniasis?. Trends Parasitol..

[22] Petersen C.A. (2009). Leishmaniasis, an emerging disease found in companion animals in the United States. Top. Companion Anim. Med..

[23] Grevot A., Jaussaud Hugues P., Marty P., Pratlong F., Ozon C., Haas P., Breton C., Bourdoiseau G. (2005). Leishmaniosis due to *Leishmania infantum* in a FIV and FelV positive cat with a squamous cell carcinoma diagnosed with histological, serological and isoenzymatic methods. Parasite.

[24] Pennisi M.G., Hartmann K., Lloret A., Addie D., Belak S., Boucraut-Baralon C., Egberink H., Frymus T., Gruffydd-Jones T., Hosie M.J. (2013). Leishmaniosis in cats: ABCD guidelines on prevention and management. J. Feline Med. Surg..

[25] Pereira A., Maia C. (2021). *Leishmania* infection in cats and feline leishmaniosis: An updated review with a proposal of a diagnosis algorithm and prevention guidelines. Curr. Res. Parasitol. Vector-Borne Dis..

[26] Pereira A., Parreira R., Cristovao J.M., Castelli G., Bruno F., Vitale F., Campino L., Maia C. (2020). Phylogenetic insights on *Leishmania* detected in cats as revealed by nucleotide sequence analysis of multiple genetic markers. Infect. Genet. Evol..

[27] Del Rio L., Chitimia L., Cubas A., Victoriano I., De la Rua P., Gerrikagoitia X., Barral M., Munoz-Garcia C.I., Goyena E., Garcia-Martinez D. (2014). Evidence for widespread *Leishmania infantum* infection among wild carnivores in *L. infantum* periendemic northern Spain. Prev. Vet. Med..

[28] Pennisi M.G., Venza M., Reale S., Vitale F., Lo Giudice S. (2004). Case report of leishmaniasis in four cats. Vet. Res. Commun..

[29] Figueiredo F.B., Bonna I.C., Nascimento L.D., Costa T., Baptista C., Pacheco T.M., Amendoeira M.R., Madeira Mde F. (2009). Serological evaluation for detection of anti-*Leishmania* antibodies in dogs and cats in the district of Santa Rita de Cassia, municipality of Barra Mansa, State of Rio de Janeiro. Rev. Soc. Bras. Med. Trop..

[30] Braga A.R., Langoni H., Lucheis S.B. (2014). Evaluation of canine and feline leishmaniasis by the association of blood culture, immunofluorescent antibody test and polymerase chain reaction. J. Venom. Anim. Toxins Incl. Trop. Dis..

[31] Trainor K.E., Porter B.F., Logan K.S., Hoffman R.J., Snowden K.F. (2010). Eight cases of feline cutaneous leishmaniasis in Texas. Vet. Pathol..

[32] Iatta R., Furlanello T., Colella V., Tarallo V.D., Latrofa M.S., Brianti E., Trerotoli P., Decaro N., Lorusso E., Schunack B. (2019). A nationwide survey of *Leishmania infantum* infection in cats and associated risk factors in Italy. PLoS Negl. Trop. Dis..

[33] Lachaud L., Marchergui-Hammami S., Chabbert E., Dereure J., Dedet J.P., Bastien P. (2002). Comparison of six PCR methods using peripheral blood for detection of canine visceral leishmaniasis. J. Clin. Microbiol..

[34] Singh S. (2006). New developments in diagnosis of leishmaniasis. Indian J. Med. Res..

[35] Ferroglio E., Centaro E., Mignone W., Trisciuoglio A. (2007). Evaluation of an ELISA rapid device for the serological diagnosis of *Leishmania infantum* infection in dog as compared with immunofluorescence assay and Western blot. Vet. Parasitol..

[36] Ferroglio E., Trisciuoglio A., Gastaldo S., Mignone W., Delle Piane M. (2002). Comparison of ELISA, IFAT and Western blot for the serological diagnosis of *Leishmania infantum* infection in dog. Parassitologia.

[37] Zuur A., Ieno E., Walker N., Saveliev A., Smith G. (2009). Mixed Effects Models and Extensions in Ecology with R.

[38] R Core Team (2015). A Language and Environment for Statistical Computing.

[39] Sarkari B., Hatam G.R., Adnani S.J., Asgari Q. (2009). Seroprevalence of feline leishmaniasis in areas of Iran where *Leishmania infantum* is endemic. Ann. Trop. Med. Parasitol..

[40] Ayllon T., Tesouro M.A., Amusategui I., Villaescusa A., Rodriguez-Franco F., Sainz A. (2008). Serologic and molecular evaluation of *Leishmania infantum* in cats from Central Spain. Ann. N. Y. Acad. Sci..

[41] Diakou A., Papadopoulos E., Lazarides K. (2009). Specific anti-*Leishmania* spp. antibodies in stray cats in Greece. J. Feline Med. Surg..

[42] Pasa S., Tetik Vardarli A., Erol N., Karakus M., Toz S., Atasoy A., Balcioglu I.C., Emek Tuna G., Ermis O.V., Ertabaklar H. (2015). Detection of *Leishmania major* and *Leishmania tropica* in domestic cats in the Ege Region of Turkey. Vet. Parasitol..

[43] Chatzis M.K., Leontides L., Athanasiou L.V., Papadopoulos E., Kasabalis D., Mylonakis M., Rallis T., Koutinas A.F., Andreadou M., Ikonomopoulos J. (2014). Evaluation of indirect immunofluorescence antibody test and enzyme-linked immunosorbent assay for the diagnosis of infection by *Leishmania infantum* in clinically normal and sick cats. Exp. Parasitol..

[44] Hervas J., Chacon-Manrique de Lara F., Lopez J., Gomez-Villamandos J.C., Guerrero M.J., Moreno A. (2001). Granulomatous (pseudotumoral) iridociclitis associated with leishmaniasis in a cat. Vet. Rec..

[45] Hervas J., Chacon M.D.L.F., Sanchez-Isarria M.A., Pellicer S., Carrasco L., Castillo J.A., Gomez-Villamandos J.C. (1999). Two cases of feline visceral and cutaneous leishmaniosis in Spain. J. Feline Med. Surg..

[46] Maia C., Gomes J., Cristovao J., Nunes M., Martins A., Rebelo E., Campino L. (2010). Feline *Leishmania* infection in a canine leishmaniasis endemic region, Portugal. Vet. Parasitol..

[47] Martin-Sanchez J., Acedo C., Munoz-Perez M., Pesson B., Marchal O., Morillas-Marquez F. (2007). Infection by Leishmania infantum in cats: Epidemiological study in Spain. Vet. Parasitol..

[48] Spada E., Proverbio D., Migliazzo A., Della Pepa A., Roberta Perego R., Giorgi B.D.G. (2013). Serological and Molecular Evaluation of *Leishmania infantum* Infection in Stray Cats in a Nonendemic Area in Northern Italy. ISRN Parasitol..

[49] Rufenacht S., Sager H., Muller N., Schaerer V., Heier A., Welle M.M., Roosje P.J. (2005). Two cases of feline leishmaniosis in Switzerland. Vet. Rec..

[50] Poli A., Abramo F., Barsotti P., Leva S., Gramiccia M., Ludovisi A., Mancianti F. (2002). Feline leishmaniosis due to *Leishmania* infantum in Italy. Vet. Parasitol..

[51] Pennisi M.G. (2015). Leishmaniosis of companion animals in Europe: An update. Vet. Parasitol..

[52] Vita S., Santori D., Aguzzi I., Petrotta E., Luciani A. (2005). Feline leishmaniasis and ehrlichiosis: Serological investigation in Abruzzo region. Vet. Res. Commun..

[53] Cardoso L., Lopes A.P., Sherry K., Schallig H., Solano-Gallego L. (2010). Low seroprevalence of *Leishmania infantum* infection in cats from northern Portugal based on DAT and ELISA. Vet. Parasitol..

[54] Duarte A., Castro I., Pereira da Fonseca I.M., Almeida V., Madeira de Carvalho L.M., Meireles J., Fazendeiro M.I., Tavares L., Vaz Y. (2010). Survey of infectious and parasitic diseases in stray cats at the Lisbon Metropolitan Area, Portugal. J. Feline Med. Surg..

[55] Ayllon T., Diniz P.P., Breitschwerdt E.B., Villaescusa A., Rodriguez-Franco F., Sainz A. (2012). Vector-borne diseases in client-owned and stray cats from Madrid, Spain. Vector Borne Zoonotic Dis..

[56] Fernandez-Perez F.J., Gomez-Munoz M.T., Mendez S., Alunda J.M. (2003). *Leishmania*-specific lymphoproliferative responses and IgG1/IgG2 immunodetection patterns by Western blot in asymptomatic, symptomatic and treated dogs. Acta Trop..

[57] Ozon C., Marty P., Pratlong F., Breton C., Blein M., Lelievre A., Haas P. (1998). Disseminated feline leishmaniosis due to *Leishmania infantum* in Southern France. Vet. Parasitol..

[58] Persichetti M.F., Solano-Gallego L., Vullo A., Masucci M., Marty P., Delaunay P., Vitale F., Pennisi M.G. (2017). Diagnostic performance of ELISA, IFAT and Western blot for the detection of anti-*Leishmania infantum* antibodies in cats using a Bayesian analysis without a gold standard. Parasit. Vectors.

[59] Otranto D., Napoli E., Latrofa M.S., Annoscia G., Tarallo V.D., Greco G., Lorusso E., Gulotta L., Falsone L., Basano F.S. (2017). Feline and canine leishmaniosis and other vector-borne diseases in the Aeolian Islands: Pathogen and vector circulation in a confined environment. Vet. Parasitol..

[60] Dedola C., Zobba R., Varcasia A., Visco S., Alberti A., Pipia A.P., Scala A., Pinna Parpaglia M.L. (2018). Serological and molecular detection of *Leishmania infantum* in cats of Northern Sardinia, Italy. Vet. Parasitol. Reg. Stud. Rep..

[61] Rocha A., Moreno B.F.S., Cabral A.D., Louzeiro N.M., Miranda L.M., Santos V., Costa F.B., Nogueira R.M.S., Marcili A., Speranca M.A. (2019). Diagnosis and epidemiology of *Leishmania infantum* in domestic cats in an endemic area of the Amazon region, Brazil. Vet. Parasitol..

[62] Mohebali M., Malmasi A., Khodabakhsh M., Zarei Z., Akhoundi B., Hajjaran H., Azarm A. (2017). Feline leishmaniosis due to *Leishmania infantum* in Northwest Iran: The role of cats in endemic areas of visceral leishmaniosis. Vet. Parasitol. Reg. Stud. Rep..

[63] Millan J., Zanet S., Gomis M., Trisciuoglio A., Negre N., Ferroglio E. (2011). An investigation into alternative reservoirs of canine leishmaniasis on the endemic island of Mallorca (Spain). Transbound. Emerg. Dis..

[64] Ruiz-Fons F., Ferroglio E., Gortazar C. (2013). *Leishmania* infantum in free-ranging hares, Spain, 2004–2010. Eurosurveillance.

[65] Zanet S., Sposimo P., Trisciuoglio A., Giannini F., Strumia F., Ferroglio E. (2014). Epidemiology of *Leishmania infantum*, *Toxoplasma gondii*, and *Neospora caninum* in *Rattus rattus* in absence of domestic reservoir and definitive hosts. Vet. Parasitol..

[66] Ferroglio E., Maroli M., Gastaldo S., Mignone W., Rossi L. (2005). Canine leishmaniasis, Italy. Emerg. Infect. Dis..

[67] Biglino A., Bolla C., Concialdi E., Trisciuoglio A., Romano A., Ferroglio E. (2010). Asymptomatic *Leishmania* infantum infection in an area of northwestern Italy (Piedmont region) where such infections are traditionally nonendemic. J. Clin. Microbiol..

[68] Ferroglio E., Romano A., Trisciuoglio A., Poggi M., Ghiggi E., Sacchi P., Biglino A. (2006). Characterization of *Leishmania infantum* strains in blood samples from infected dogs and humans by PCR-RFLP. Trans. R. Soc. Trop. Med. Hyg..

[69] Sundar S., Singh O.P. (2018). Molecular Diagnosis of Visceral Leishmaniasis. Mol. Diagn. Ther..

[70] Akhoundi M., Downing T., Votypka J., Kuhls K., Lukes J., Cannet A., Ravel C., Marty P., Delaunay P., Kasbari M. (2017). *Leishmania* infections: Molecular targets and diagnosis. Mol. Asp. Med..

[71] Nardy S.C., Estangari R.F. (2007). Métodos de diagnóstico da leishmaniose visceral canina. Rev. Clín. Vet..

[72] Antinori S., Calattini S., Longhi E., Bestetti G., Piolini R., Magni C., Orlando G., Gramiccia M., Acquaviva V., Foschi A. (2007). Clinical use of polymerase chain reaction performed on peripheral blood and bone marrow samples for the diagnosis and monitoring of visceral leishmaniasis in HIV-infected and HIV-uninfected patients: A single-center, 8-year experience in Italy and review of the literature. Clin. Infect. Dis..

[73] Mortarino M., Franceschi A., Mancianti F., Bazzocchi C., Genchi C., Bandi C. (2004). Quantitative PCR in the diagnosis of *Leishmania*. Parassitologia.

[74] Gramiccia M. (2011). Recent advances in leishmaniosis in pet animals: Epidemiology, diagnostics and anti-vectorial prophylaxis. Vet. Parasitol..

[75] Tabar M.D., Altet L., Francino O., Sanchez A., Ferrer L., Roura X. (2008). Vector-borne infections in cats: Molecular study in Barcelona area (Spain). Vet. Parasitol..

[76] Asfaram S., Fakhar M., Teshnizi S.H. (2019). Is the cat an important reservoir host for visceral leishmaniasis? A systematic review with meta-analysis. J. Venom. Anim. Toxins Incl. Trop. Dis..

[77] Pennisi M.G., Persichetti M.F. (2018). Feline leishmaniosis: Is the cat a small dog?. Vet. Parasitol..

[78] Weese J.S., Evason M. (1998). Infectious Diseases of the Dog and Cat.

[79] Roura X., Peters I.R., Altet L., Tabar M.D., Barker E.N., Planellas M., Helps C.R., Francino O., Shaw S.E., Tasker S. (2010). Prevalence of hemotropic *mycoplasmas* in healthy and unhealthy cats and dogs in Spain. J. Vet. Diagn. Investig..

[80] Latrofa M.S., Iatta R., Toniolo F., Furlanello T., Ravagnan S., Capelli G., Schunack B., Chomel B., Zatelli A., Mendoza-Roldan J. (2020). A molecular survey of vector-borne pathogens and haemoplasmas in owned cats across Italy. Parasit. Vectors.

[81] Bezerra J.A.B., Oliveira I., Yamakawa A.C., Nilsson M.G., Tomaz K.L.R., Oliveira K.D.S., Rocha C.S.D., Calabuig C.I.P., Fornazari F., Langoni H. (2019). Serological and molecular investigation of *Leishmania* spp. infection in cats from an area endemic for canine and human leishmaniasis in Northeast Brazil. Rev. Bras. Parasitol. Vet..

[82] Elmahallawy E.K., Alkhaldi A.A.M., Saleh A.A. (2021). Host immune response against leishmaniasis and parasite persistence strategies: A review and assessment of recent research. Biomed. Pharmacother..

[83] Elmahallawy E.K., Alkhaldi A.A.M. (2021). Insights into *Leishmania* Molecules and Their Potential Contribution to the Virulence of the Parasite. Vet. Sci..

[84] Maroli M., Pennisi M.G., Di Muccio T., Khoury C., Gradoni L., Gramiccia M. (2007). Infection of sandflies by a cat naturally infected with *Leishmania infantum*. Vet. Parasitol..

[85] Mendonca I.L., Batista J.F., Lopes K., Magalhaes Neto F., Alcantara D.S., Merigueti Y., Costa C.H.N. (2020). Infection of *Lutzomyia longipalpis* in cats infected with *Leishmania infantum*. Vet. Parasitol..

[86] Baneth G., Greene C.E. (2006). Leishmaniasis. Infectious Diseases of the Dog and Cat.

