# Supplementary material for: Feline Leishmaniosis in Northwestern Italy: Current Status and Zoonotic Implications
Source: Vet Sci. 2021 Oct 2;8(10):215. doi: 10.3390/vetsci8100215 (PMC8539510; doi:10.3390/vetsci8100215)
Supplement: Supplementary file 1 [file vetsci-08-00215-s001.zip › vetsci-1307945-supplementary.pdf]

veterinary sciences

MDPI

Supporting Material

Feline Leishmaniosis in Northwestern Italy: Current Status and Implications

Ehab Kotb Elmahallawy [1,2,3*] , Stefania Zanet [3], Marco Poggi [3], Khalaf F Alsharif [4], Ahmad Agil [2], Anna Trisciuoglio [3] and Ezio Ferroglio [3]

Table S1. The full details of the study cohort for each of the enrolled cat's sex, age, breed and living habits.

		Enrolled cats
Sex	M	31
	M neutered	176
	F	27
	F spayed	112
Age	12-36 months	114
	3 - 10 yrs	178
	≥10 yrs	54
Breed	European	315
	British Short Hair	7
	Maine Coon	12
	Norwegian Forest Cat	3
	Persian	9
Living habits	Inside only	163
	Nights outside	59
	Outside only	124
